# *Kluyveromyces lactis* Hydrolysate Enhances Growth Performance and Reduces Diarrhea Rate by Modulating Immune Function and Regulating Gut Microbiota in Weaned Piglets

**DOI:** 10.3390/microorganisms14071440

**Published:** 2026-06-30

**Authors:** Yuyang Fan, Chenggang Yin, Xinyue Jiang, Lei Xu, Ge Gao, Dongxu Ming, Yanpin Li, Wenjuan Sun, Xilong Li, Yu Pi

**Affiliations:** Key Laboratory of Feed Biotechnology of the Ministry of Agriculture and Rural Affairs, Institute of Feed Research, Chinese Academy of Agricultural Sciences, Beijing 100081, China; w18832417225@163.com (Y.F.); ycg0701@126.com (C.Y.); j15613565090@163.com (X.J.); xlei0611@163.com (L.X.); 82101192355@caas.cn (G.G.); mingdongxu@caas.cn (D.M.); liyanpin@caas.cn (Y.L.); sunwenjuan@caas.cn (W.S.)

**Keywords:** *Kluyveromyces lactis* hydrolysate, diarrhea, immunity, intestinal health, gut microbiota, weaned piglets

## Abstract

This study assessed the effect of dietary supplementation with *Kluyveromyces lactis* (*K. lactis*) hydrolysate (HKL) on growth performance, apparent nutrient digestibility, systemic immune–antioxidant status, and fecal microbiota in weaned piglets. A total of fifty-four piglets, with an initial body weight of 6.07 ± 0.086 kg and age of 25 ± 1 days, were randomly assigned to three dietary treatments over 28 days (6 replicates per treatment; 3 piglets per replicate): a control diet (CON), CON supplemented with 5 g/kg HKL (HKL1), or CON supplemented with 10 g/kg HKL (HKL2). Throughout the trial, growth performance was monitored, apparent total tract digestibility of nutrients was determined, serum samples were collected for immune and antioxidant assessments, and fecal samples were gathered for microbiota analysis. The results indicated that compared to the CON group, both HKL1 and HKL2 groups exhibited improved growth performance, as evidenced by increased average daily feed intake (ADFI) from day 0 to 28 (*p* < 0.05). Furthermore, HKL2 significantly enhanced average daily gain (ADG) from day 0 to 14 and reduced the feed-to-gain ratio (F: G) during the same period (*p* < 0.05). Diarrhea incidence was markedly decreased by HKL supplementation at both day 1–14 and day 15–28 (*p* < 0.001). HKL supplementation increased the apparent digestibility of dry matter, ash, calcium, and phosphorus (*p* < 0.05). On day 14, serum total protein and immunoglobulin A (IgA) levels were elevated, while malondialdehyde (MDA) levels were reduced in HKL-supplemented piglets (*p* < 0.05). By day 28, serum immunoglobulin G (IgG) concentrations, as well as superoxide dismutase (SOD) and glutathione peroxidase (GSH-Px) activities, were increased, while interleukin-6 (IL-6) levels were decreased in HKL-supplemented piglets (*p* < 0.05), suggesting HKL possesses immunomodulatory and antioxidant regulatory capacities. HKL also enriched several health-associated commensal bacteria, including [*Eubacterium*]_*xylanophilum*_group, unclassified_f_Peptostreptococcaceae, *Candidatus Saccharimonas*, Erysipelotrichaceae_UCG-003, and *Negativibacillus*, suggesting a microbiota-modulatory effect in weaned piglets. These results indicate that dietary supplementation with HKL could improve growth performance and nutrient utilization, reduce post-weaning diarrhea, and promote a more favorable immune–antioxidant status and microbial profile in weaned piglets, with the 10 g/kg dosage demonstrating greater overall efficacy. These findings provide a theoretical basis for the development of novel *K. lactis* products and the application of *K. lactis* hydrolysate in weaned piglets.

## 1. Introduction

In modern swine production, weaning is a critical transition period that exposes piglets to multifactorial stressors, including abrupt dietary changes, maternal separation, and environmental adaptations. These stressors disrupt the homeostasis of the gastrointestinal tract, often leading to “post-weaning syndrome,” characterized by growth retardation, anorexia, and diarrhea [1]. The underlying pathophysiology involves an immature digestive enzyme system and an underdeveloped immune system. Consequently, weaning stress damages the intestinal mucosal barrier and increases intestinal permeability, facilitating pathogen translocation and triggering systemic inflammation and oxidative stress [2,3]. This cascade of events—where gut dysbiosis exacerbates epithelial damage, leading to immune activation and further oxidative injury—ultimately impairs nutrient absorption and growth performance [2,4,5]. Following the restrictions or withdrawal of antibiotic growth promoters in many regions, there is an urgent need to identify effective alternatives that can target these specific biological mechanisms to restore intestinal health and resilience in weaned piglets.

Yeast hydrolysates, derived from the enzymatic hydrolysis of yeast cells, have emerged as promising functional feed additives. Unlike whole yeast cells, hydrolysates contain high concentrations of functional nucleotides, bioactive peptides, and cell wall polysaccharides (such as β-glucans and mannan-oligosaccharides), and their byproducts are known for their highly digestible protein, nucleotide, and amino acid (glutamic acid) contents [6,7]. These components may exert beneficial effects through several interconnected mechanisms. Previous studies have suggested that nucleotides and small peptides can support intestinal development and tissue repair, thereby contributing to improved nutrient utilization [6,8]. In addition, bioactive peptides and yeast cell wall components have been reported to modulate immune responses and influence cytokine production, which may help alleviate inflammatory stress and support humoral immunity [6,9,10]. Collectively, these findings suggest that yeast hydrolysates have the potential to promote intestinal health and immune function. However, intestinal morphology, tight junction integrity, barrier function, and immune-cell interactions were not directly evaluated in the present study [11,12]. Consequently, dietary supplementation with yeast hydrolysates has been shown to improve growth performance, reduce diarrhea incidence, and enhance apparent nutrient digestibility in weaned piglets [7,13,14]. Furthermore, these additives alleviate weaning-associated oxidative stress by increasing superoxide dismutase (SOD) and glutathione peroxidase (GSH-Px) activities while decreasing malondialdehyde (MDA) levels [15].

Despite the well-documented benefits of Saccharomyces cerevisiae-based products, the efficacy of yeast hydrolysates is inherently strain-dependent due to variations in cell wall composition and intracellular protein profiles among yeast species. *Kluyveromyces lactis* (*K. lactis*) is a “Generally Recognized As Safe” (GRAS) yeast strain with a unique metabolic profile and high protein content [16], yet its potential application in animal nutrition remains largely unexplored. Furthermore, determining the optimal dietary inclusion level is critical for maximizing efficacy while ensuring economic viability. Based on previous studies evaluating yeast derivatives in swine diets, which reported positive outcomes within the range of 2.5 to 15 g/kg [15,17,18], we selected two inclusion levels: 5 g/kg, which represents a standard supplemental dose to assess basic efficacy, and 10 g/kg, which was included to examine whether a higher supplementation level could provide additional benefits within the practical range reported in previous studies. Therefore, the novelty of the present study lies in evaluating the effects of *K. lactis* hydrolysate, a relatively unexplored yeast-derived product in swine nutrition, on growth performance, nutrient digestibility, immune-antioxidant status, inflammatory responses, and gut microbiota composition in weaned piglets. In addition, two supplementation levels (5 and 10 g/kg) were evaluated to investigate potential dose-dependent responses and identify an effective dietary inclusion level.

## 2. Materials and Methods

The experimental trial was conducted at the Tianpeng Experimental Farm in Langfang, Hebei Province, China, from April to June 2025. The animal protocol for this research was approved by the Animal Care and Use Committee of the Institute of Feed Research of the Chinese Academy of Agricultural Sciences (IFR-CAAS20250808).

### 2.1. Preparation of K. lactis Hydrolysate

The *K. lactis* used in the present study was isolated from decaying wood by the pig nutrition and feed innovation team of the Feed Research Institute, Chinese Academy of Agricultural Sciences, and is currently deposited with the China General Microbiological Culture Collection Center (CGMCC No. 30910).

The strains were first revived and cultured in yeast extract peptone dextrose (YPD) medium (Beijing Kulaibo Technology Co., Ltd., Beijing, China) for 3 d. The seed culture medium consisted of glucose (20 g/L), yeast extract (10 g/L), and peptone (20 g/L). Subsequently, fermentation was conducted in a stirred-tank bioreactor (Thermo Fisher Scientific, Waltham, MA, USA) with a working volume of 3 L. The fermentation medium contained (NH_4_)_2_SO_4_ (5 g/L), KH_2_PO_4_ (1 g/L), MgSO_4_ (0.2 g/L), CaCl_2_ (0.1 g/L), and reed straw (80 g/L). The culture conditions were maintained at 30 °C with an agitation speed of 150 rpm, an initial pH of 7.0, and an inoculum size of 2%. Aeration was continuously supplied using an air blower, and the dissolved oxygen level was maintained at 30% saturation throughout the fermentation process. Fermentation time lasts for 3 days. The obtained *K. lactis* cells were subsequently used for the preparation of hydrolysates.

The preparation method for *K. lactis* hydrolysate is as follows: The *K. lactis* culture was concentrated to a dry weight percentage of 10–12%. The initial pH value was measured using a pH meter (PB-10, Sartorius, Göttingen, Germany) and adjusted to 5.0 (using phosphate-buffered solution). Then, 0.3% (*w*/*w*) of yeast cell wall lytic enzyme A (a complex protease) was added to the culture, and the mixture was enzymatically hydrolyzed for 3 h at 45 °C. This enzyme primarily hydrolyzes protein bonds selectively, disrupting protein–polysaccharide complexes in the cell wall and initially opening the cell structure. After the enzymatic hydrolysis, the pH was checked and adjusted to 9.0 (using sodium hydroxide solution), followed by the addition of 0.1% (*w*/*w*) of yeast cell wall lytic enzyme B (cellulase B), and the mixture was further hydrolyzed for 3 h at 55 °C. This enzyme efficiently degrades β-glucans and cellulose, further breaking down the cell wall and releasing intracellular active components. Both the yeast cell wall lytic enzyme A and yeast cell wall lytic enzyme B are provided by Inner Mongolia Keweibo Biotechnology Co., Ltd. (Chifeng, China). Once the enzymatic hydrolysis was complete, the moisture was removed using spray drying technology to obtain the final product of *K. lactis* hydrolysate. The *K. lactis* hydrolysis effect was evaluated by microscopy, the dilution coating plate counting method, and the determination of nutritional components, mannan, β-glucan, and free amino acids.

### 2.2. Microscopic Examination

To examine *K. lactis*, the fermentation broth was centrifuged at 5000 rpm for 10 min, and a portion of the supernatant was removed. The remaining sample was thoroughly mixed, and a small aliquot was placed on a microscope slide for observation and image acquisition following the previously described method [19]. *K. lactis* hydrolysate was prepared by treating an equivalent amount of *K. lactis* using the aforementioned hydrolysis method and examined under the same conditions. Images were obtained using a light microscope equipped with a 10× eyepiece and a 6× objective lens.

### 2.3. Spread Plate Method

YPD solid plates were prepared by adding 2% agar powder to YPD medium. The mixture was sterilized at 121 °C for 15 min, poured into plates, and cooled for later use. Equivalent volumes of *K. lactis* suspensions before and after cell wall disruption were serially diluted with sterile water. A 100 μL aliquot of each dilution was pipetted onto the center of the plates and spread evenly over the medium surface using a sterile spreader. The plates were inverted and incubated at 30 °C for 24~48 h. Visible single colonies were then used for colony counting.

### 2.4. Determination of Nutritional Components, Mannan, β-Glucan, and Free Amino Acids

The *K. lactis* suspensions before and after hydrolysis were clarified by centrifugation at 5000× *g* for 10 min to remove precipitates, and the supernatants were collected for amino acid analysis. Subsequently, amino acid analysis was performed using an HPLC system (LC-20AT, Shimadzu, Kyoto, Japan) following pre-column derivatization with o-phthalaldehyde (OPA), as previously described [20]. Separation was achieved on a C18 reversed-phase column (4.6 × 250 mm, 5 μm) using gradient elution. Individual amino acids were identified and quantified by comparison with authenticated amino acid standards.

HKL samples were analyzed for dry matter (DM; method 930.15), crude protein (CP; N × 6.25; method 968.06), and ether extract (method 954.02) according to Association of Official Analytical Chemists (AOAC) procedures [21]. Trichloroacetic acid-soluble protein (TCA-SP) levels were measured as previously described by Ovissipour et al. (2009) [22]. The contents of mannan and β-glucan were quantified according to the previous method [23].

### 2.5. Experimental Design and Animal Management

A total of 54 healthy weaned piglets (Duroc × Landrace × Yorkshire) with similar initial body weight (6.07 ± 0.086 kg) and age (25 ± 1 day), and with no history of disease, were selected for the trial. Piglets were balanced for sex (an equal ratio of barrows to gilts) and randomly assigned to three dietary treatments using a completely randomized design. Each treatment consisted of six replicates (pens), with three piglets per replicate (*n* = 6). The treatments consisted of a basal diet (control; CON) and two experimental diets containing HKL at 5 g/kg (HKL1) or 10 g/kg (HKL2). Piglets in the CON group were fed a corn-soybean meal basal diet, whereas the HKL1 and HKL2 groups received the basal diet supplemented with 5 g/kg and 10 g/kg of *K. lactis* hydrolysate, respectively. The experimental diets were formulated to be isocaloric and isonitrogenous by replacing equivalent amounts of corn and soybean meal in the basal diet. The inclusion levels of *K. lactis* hydrolysate were determined based on previously published doses of *Kluyveromyces fragilis* hydrolysate used in weaned piglets [15]. All diets were formulated to meet or exceed the nutrient requirements recommended by the National Research Council (NRC, 2012) [24]. The ingredient composition and analyzed nutrient levels of the diet are presented in Table 1. No antibiotic growth promoters were included, and diets were provided in mash form. The experimental period lasted 28 days. Throughout the experiment, piglets had ad libitum access to feed and water. Piglets were housed in slatted-floor pens (1.7 m × 1.5 m per pen). Lighting and ventilation were checked daily. Ventilation was provided by variable-speed fans in combination with natural and artificial light. Ambient temperature was regulated using an automatic environmental control system. Disinfection and vaccination were performed according to standard farm management procedures.

### 2.6. Growth Performance and Diarrhea Incidence Measurements

Each piglet was individually weighed on days 0, 14, and 28. Feed intake was recorded per replicate pen on days 14 and 28. Average daily gain (ADG), average daily feed intake (ADFI), and the feed-to-gain ratio (F: G) were calculated from body weight and feed intake data. Fecal scores were assessed once daily in the morning by visual inspection using the following scale: 0 = hard, dry, and granular; 1 = hard and well-formed; 2 = soft, moist, and formed; 3 = soft and unformed; 4 = watery. Fecal scores of 3 or 4 were defined as diarrhea [25]. Diarrhea rate (%) was calculated as: Diarrhea rate (%) = [number of diarrhea cases/(total number of piglets × total experimental days)] × 100.

### 2.7. Samples Collection

Diet samples were collected during feed manufacture at the packing outlet, reduced by quartering, ground, passed through a 40-mesh sieve, and stored in sealed bags at room temperature for nutrient analysis. On days 14 and 28, one pig per pen was randomly selected for sampling. A total of 10 mL of blood was collected from the anterior vena cava in the morning before feeding into blood collection tubes (Vacutainer tubes without anticoagulant). The serum was separated by centrifugation (3000× *g*, 10 min, 4 °C) and preserved at −20 °C for later analysis of immune, antioxidant, and inflammatory indices. Fresh fecal samples were obtained from each replicate on days 26–28, pooled by replicate, and oven-dried at 65 °C for 48 h. The dried samples were equilibrated at room temperature for 24 h, ground, passed through a 40-mesh sieve, and stored for apparent digestibility determination. Additionally, on days 14 and 28, one piglet was randomly selected from each pen, and approximately 2 g of fresh feces was collected into sterile tubes, snap-frozen in liquid nitrogen, and stored at −80 °C for microbiota analysis.

### 2.8. Apparent Total Tract Nutrients Digestibility Measurement and Calculation

To determine the apparent total tract digestibility (ATTD) of nutrients, fecal samples were collected twice daily during the final three days of the trial (days 26–28). Individual fecal samples were pooled within animals and homogenized before analysis. Diet and fecal samples were analyzed for dry matter (DM; method 930.15), crude protein (CP; N × 6.25; method 968.06), crude fiber (method 991.43), calcium (Ca; method 984.01), phosphorus (P; method 965.17), ether extract (method 954.02), ash (method 942.05), and acid-insoluble ash (AIA; method 942.05) according to AOAC procedures [21]. Gross energy (GE) was determined using an adiabatic bomb calorimeter (Parr 1281, Parr Instrument Company, Moline, IL, USA). AIA was used as an internal marker to calculate ATTD using the indicator method. The formula for calculating the ATTD is as follows:

ATTD = [1 − (A_feed_ × N_feces_)/(N_feed_ ×A_feces_)] × 100

where A_feed_ = Content of AIA in feed (%); A_feces_ = Content of AIA in feces (%); N_feed_ = Content of a certain nutrient in feed (%); N_feces_ = Content of a certain nutrient in feces (%).

### 2.9. Serum Biochemical, Antioxidant, and Inflammation-Related Indices Determination

Serum alanine aminotransferase (ALT), aspartate aminotransferase (AST), glucose (GLU), total protein (TP), albumin (ALB), low-density lipoprotein cholesterol (LDL-C), high-density lipoprotein cholesterol (HDL-C), total cholesterol (TC), and triglycerides (TG) were determined using commercial assay kits (Maikalu Biotechnology Co., Ltd., Chengdu, China) on an automated biochemical analyzer (Erba XL-200; Erba Diagnostics, Mannheim, Germany). Serum inflammatory cytokines—including interleukin-6 (IL-6), interleukin-8 (IL-8), interleukin-10 (IL-10), interleukin-1β (IL-1β), and tumor necrosis factor-α (TNF-α)—and immunoglobulins including immunoglobulin A (IgA), immunoglobulin G (IgG), and immunoglobulin M (IgM) were quantified using Enzyme-Linked Immunosorbent Assay (ELISA) kits (Shanghai Enzyme-linked Biotechnology Co., Ltd., Shanghai, China) following the manufacturer’s instructions. In addition, serum catalase (CAT), glutathione peroxidase (GSH-Px), superoxide dismutase (SOD), malondialdehyde (MDA), and total antioxidant capacity (T-AOC) were measured using commercial assay kits (Nanjing Jiancheng Bioengineering Institute, Nanjing, China) according to the manufacturers’ instructions.

### 2.10. Fecal Microbiome Analysis

Total microbial DNA was extracted from fecal samples using a commercial kit (Omega Bio-Tek, Norcross, GA, USA) following the manufacturer’s instructions. The V3-V4 hypervariable region of the bacterial 16S rRNA gene was amplified using primers 338F (5′-ACTCCTACGGGAGGCAGCAG-3′) and 806R (5′-GGACTACHVGGGTWTCTAAT-3′) [15]. PCR products were purified using a commercial PCR clean-up kit (Axygen Biosciences, Union City, CA, USA) and subjected to paired-end sequencing on an Illumina MiSeq platform with a 2 × 250 bp configuration.

Raw reads were quality-filtered and denoised using DADA2 to infer amplicon sequence variants (ASVs), including chimera removal, under default parameters unless otherwise stated. Representative ASV sequences were taxonomically assigned in QIIME 2 (v2022.2) using a naïve Bayes classifier against the SILVA 138 reference database. Alpha-diversity indices were calculated in Mothur (version 1.3), and beta-diversity was computed based on Bray–Curtis dissimilarities using the vegan package (version 3.3.1) in R (version 3.6.2). Community-level differences were visualized by principal coordinate analysis (PCoA) based on Bray–Curtis, and statistical significance among groups was evaluated by analysis of similarities (ANOSIM; 999 permutations). Differences in relative abundances of microbiota were evaluated using the Kruskal–Wallis H test, and multiple comparisons between groups were performed using the Benjamini–Hochberg false discovery rate (FDR) correction. Bioinformatic analyses were conducted using the Majorbio Cloud Platform (https://cloud.majorbio.com/) (accessed on 9 November 2025) (Shanghai Majorbio Bio-Pharm Technology Co., Ltd., Shanghai, China).

### 2.11. Statistical Analysis

Statistical analyses were performed using SAS (version 9.4; SAS Institute Inc., Cary, NC, USA). Based on the experimental design, the experimental unit differed among the measured variables. For growth performance and nutrient digestibility, the experimental pen (*n* = 6 per treatment) served as the experimental unit, as piglets were housed and fed collectively. Data were analyzed using a one-way analysis of variance (ANOVA) under a completely randomized design (CRD). The model used was:

Y_ij_ = μ + T_i_ + e_ij_

where Y_ij_ is the observation, μ is the overall mean, T_i_ is the fixed effect of the dietary treatment (i = 1, 2, 3), and e_ij_ is the random residual error.

For growth performance, the pen was considered the experimental unit (*n* = 6). For serum biochemical, immune, antioxidant, inflammatory, and fecal microbiota analyses, one piglet was randomly selected from each pen for sampling, and the sampled piglet represented the corresponding pen (*n* = 6). The incidence of diarrhea was analyzed using the Chi-square test, with the individual piglet as the experimental unit. For all ANOVA analyses, differences among treatments were separated using Tukey’s multiple-comparison test. Statistical significance was set at *p* < 0.05, and a tendency toward a difference was considered when 0.05 ≤ *p* < 0.10.

## 3. Results

### 3.1. Evaluation of the Hydrolysis Efficiency in K. lactis

**Figure 1:**
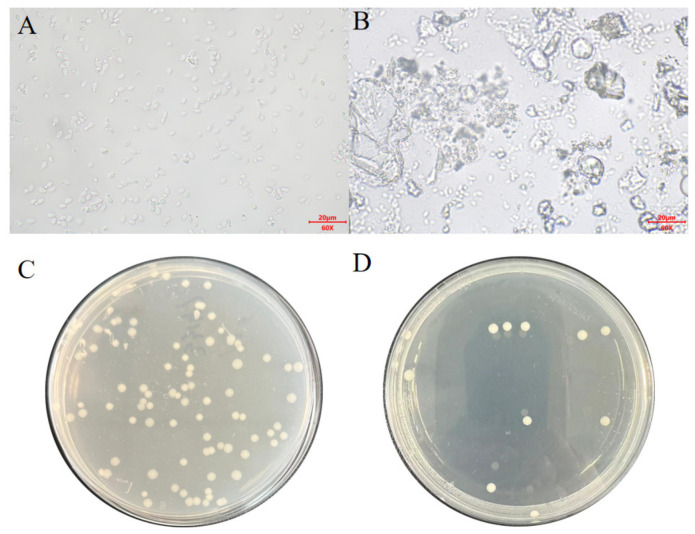
Comparison of *Kluyveromyces lactis* before and after hydrolysis. (**A**) Microscopic examination before wall perforation; (**B**) Post-wall-breaking microscopic examination; (**C**) Apply the coating on the plate before breaking the wall; (**D**) After breaking the wall, apply the plate coating.

Light microscopic examination revealed that untreated *K. lactis* cells exhibited a typical ovoid to ellipsoidal morphology, characterized by regular shapes and clearly defined boundaries. These cells appeared intact with a uniform distribution, and no obvious cellular debris was observed (Figure 1A). In contrast, following cell-wall disruption, the population of intact cells decreased markedly. The micrographs showed extensive cellular rupture and the leakage of intracellular contents, characterized by numerous irregular cell fragments, remnants of cell walls, and amorphous cytoplasmic material. These observations indicated a substantial loss of cellular structural integrity (Figure 1B).

Plate culture results further confirmed the impact of the disruption process on yeast viability. The non-disrupted samples yielded abundant and dense single colonies on solid medium, suggesting preserved viability and proliferative potential (Figure 1C). Conversely, only a few colonies were observed in the disrupted samples, indicating that the majority of cells had lost viability following cell-wall rupture and were unable to proliferate into visible colonies (Figure 1D).

As presented in Table 2, cell wall disruption significantly modified the free amino acid profile of *K. lactis*. Concentrations of aspartic acid (Asp), glutamic acid (Glu), serine (Ser), histidine (His), glycine (Gly), threonine (Thr), alanine (Ala), tyrosine (Tyr), valine (Val), methionine (Met), isoleucine (Ile), phenylalanine (Phe), and leucine (Leu) all increased significantly following disruption (*p* < 0.05). While arginine (Arg) and lysine (Lys) exhibited an upward trend (*p* < 0.10), the differences were not statistically significant (*p* > 0.05). Norvaline (Nva) levels remained unchanged by the disruption treatment (*p* > 0.05).

Table 3 presents the nutrient levels, as well as the mannan and β-glucan contents, of *K. lactis* hydrolysate. Specifically, the contents of crude fat, crude protein, and trichloroacetic acid soluble protein were 1.7%, 33.05%, and 9.19%, respectively. The mannan and β-glucan contents were 7.18% and 10.90%, respectively.

### 3.2. Effects of Dietary HKL on Growth Performance, Diarrhea Incidence, and Apparent Nutrient Digestibility in Weaned Piglets

As shown in Table 4, during days 1–14, supplementation with 10 g/kg HKL increased ADG and F: G compared with the CON group (*p* < 0.05). In addition, piglets fed 10 g/kg HKL tended to have a higher BW on day 14 (*p* = 0.089). During the overall period (days 1–28), both 5 and 10 g/kg HKL increased ADFI relative to CON (*p* < 0.05). Moreover, a trend toward increased ADG was observed in the 10 g/kg HKL group (*p* = 0.059). As presented in Table 5, dietary supplementation with 5 or 10 g/kg HKL reduced the incidence of diarrhea during both phases of the experiment (days 1–14 and days 15–28) compared with the CON group (*p* < 0.001).

**Figure 2:**
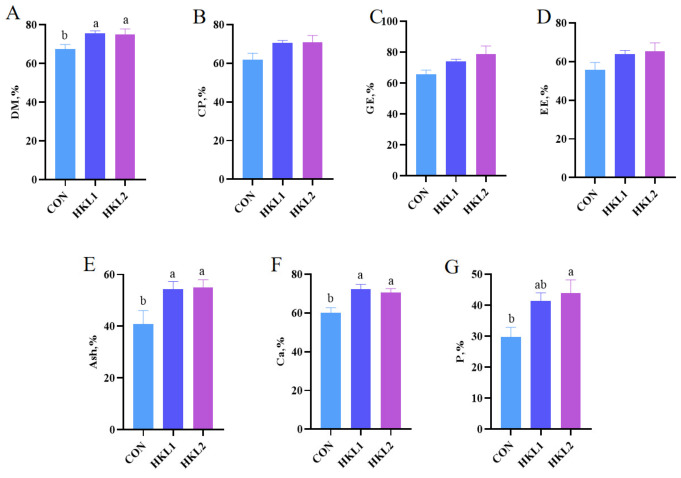
Effect of dietary *K. lactis* hydrolysate (HKL) supplementation on apparent total tract digestibility in weaned piglets. (**A**) DM, dry matter; (**B**) CP, crude protein; (**C**) GE, gross energy; (**D**) EE, ether extract; (**E**) Ash; (**F**) Ca, calcium; (**G**) P, phosphorus. CON = basal diet without additive; HKL1 = CON + HKL (5 g/kg); HKL2 = CON + HKL (10 g/kg); *n* = 6. The result is presented as mean ± standard error. ^a,b^ The value with a different superscript means a significant difference (*p* < 0.05).

As illustrated in Figure 2, compared with the CON group, supplementation with 5 or 10 g/kg HKL significantly increased the ATTD of DM, Ash, and Ca (*p* < 0.05). In addition, supplementation with 10 g/kg HKL significantly increased the ATTD of P (*p* < 0.05). However, no significant effects were observed on the ATTD of CP, GE, and EE (*p* > 0.05).

### 3.3. Effects of Dietary HKL on Serum Biochemical Indices in Weaned Piglets

**Figure 3:**
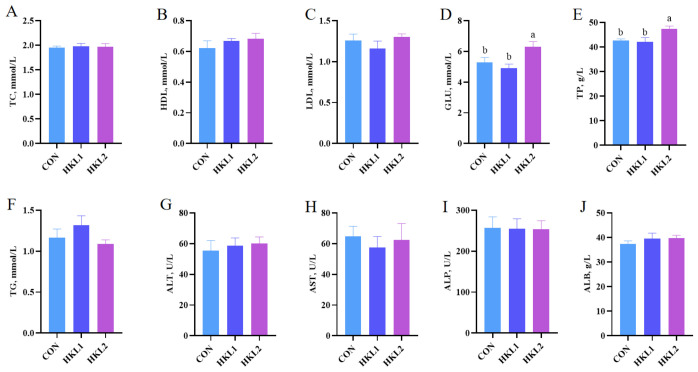

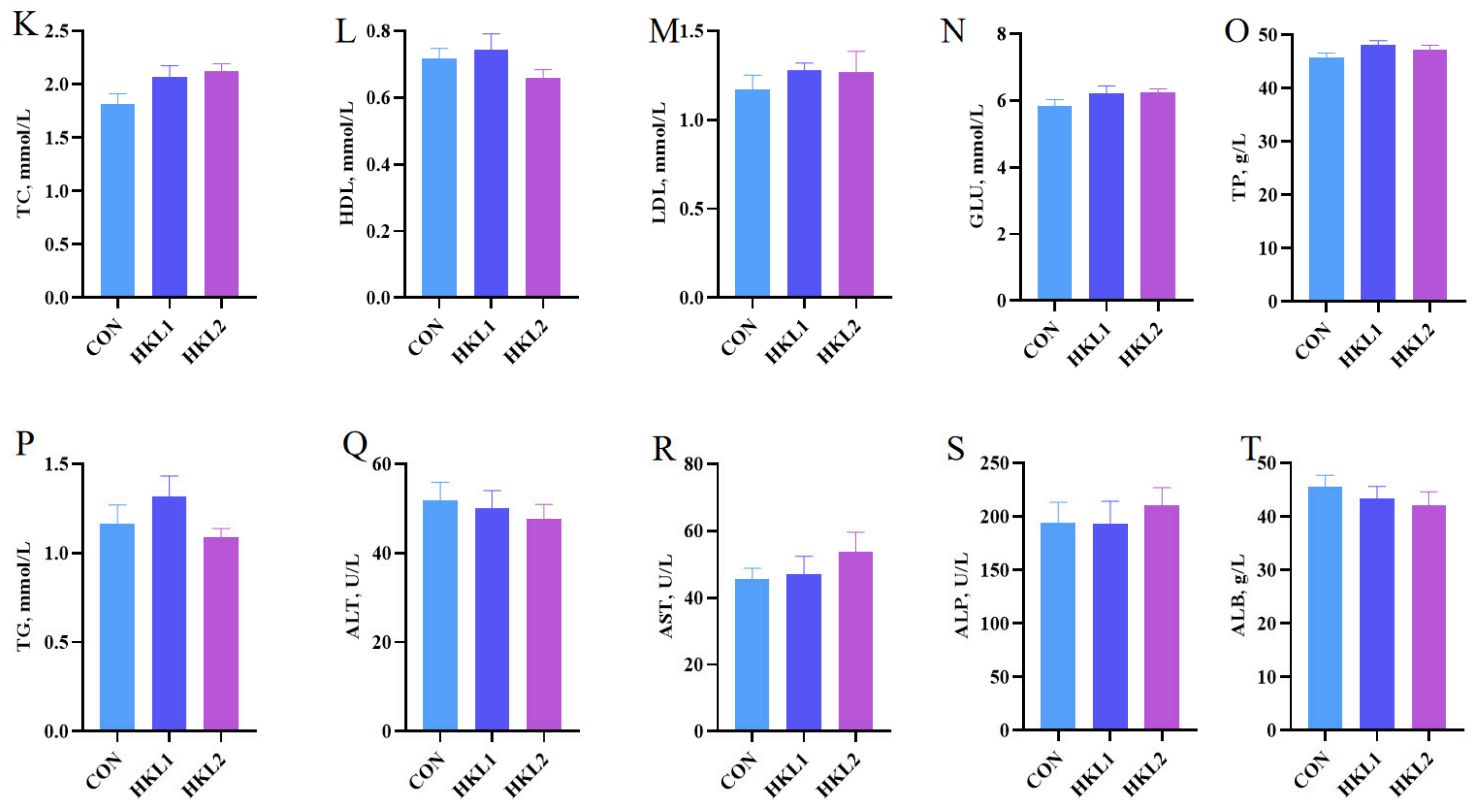
Effects of dietary *K. lactis* hydrolysate (HKL) supplementation on serum biochemical parameters in weaned piglets on day 14 (**A**–**J**) and day 28 (**K**–**T**). TC, total cholesterol (**A**,**K**); HDL-C, high-density lipoprotein cholesterol (**B**,**L**); LDL-C, low-density lipoprotein cholesterol (**C**,**M**); GLU, glucose (**D**,**N**); TP, total protein (**E**,**O**); TG, triglycerides (**F**,**P**); ALT, alanine aminotransferase (**G**,**Q**); AST, aspartate aminotransferase (**H**,**R**); ALP, alkaline phosphatase (**I**,**S**); ALB, albumin (**J**,**T**). CON = basal diet without additive; HKL1 = CON + HKL (5 g/kg); HKL2 = CON + HKL (10 g/kg). *n* = 6. The result is presented as mean ± standard error. ^a,b^ The value with a different superscript means a significant difference (*p* < 0.05).

As shown in Figure 3, compared with the CON group, supplementation with 10 g/kg HKL significantly increased serum GLU and TP concentrations on day 14 (*p* < 0.05; Figure 3D,E). However, it had no significant effect on the level of TC, HDL-C, LDL-C, TG, ALT, AST, ALP, and ALB (*p* > 0.05; Figure 3A–C,F–J). On day 28, dietary supplementation with HKL had no significant effect on blood biochemistry in weaned piglets (*p* > 0.05; Figure 3K–T).

### 3.4. Effects of Dietary HKL on Serum Antioxidant Indices in Weaned Piglets

**Figure 4:**
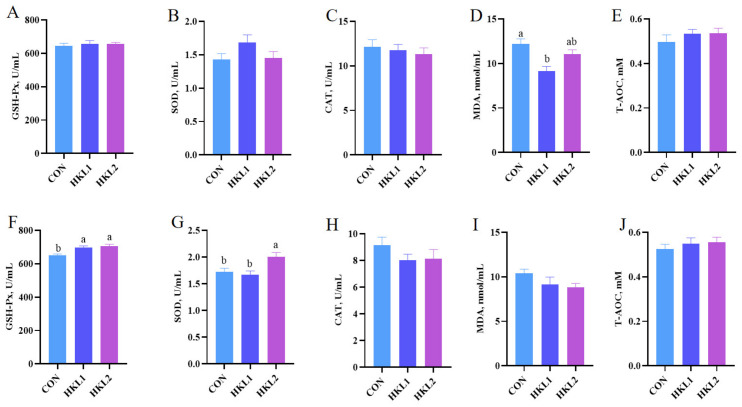
Effects of dietary *K. lactis* hydrolysate (HKL) supplementation on serum antioxidant indices in weaned piglets on day 14 and day 28. (**A**,**F**) GSH-Px, glutathione peroxidase; SOD, superoxide dismutase (**B**,**G**); CAT, catalase (**C**,**H**); MDA, malondialdehyde (**D**,**I**); T-AOC, total antioxidant capacity (**E**,**J**). CON = basal diet without additive; HKL1 = CON + HKL (5 g/kg); HKL2 = CON + HKL (10 g/kg). *n* = 6. The result is presented as mean ± standard error. ^a,b^ The value with a different superscript means a significant difference (*p* < 0.05).

As shown in Figure 4, on day 14, supplementation with 5 g/kg HKL significantly decreased serum MDA concentration compared with the CON group (*p* < 0.05; Figure 4D). However, it had no significant effect on the activity of GSH-Px, SOD, CAT, and T-AOC (*p* > 0.05; Figure 4A–C,E). On day 28, both 5 and 10 g/kg HKL significantly increased serum GSH-Px activity (*p* < 0.05; Figure 4F), and 10 g/kg HKL further increased serum SOD activity relative to CON (*p* < 0.05; Figure 4G). However, it had no significant effect on CAT activity and the level of MDA and T-AOC (*p* > 0.05; Figure 4H–J).

### 3.5. Effects of Dietary HKL on Serum Cytokines Levles in Weaned Piglets

**Figure 5:**
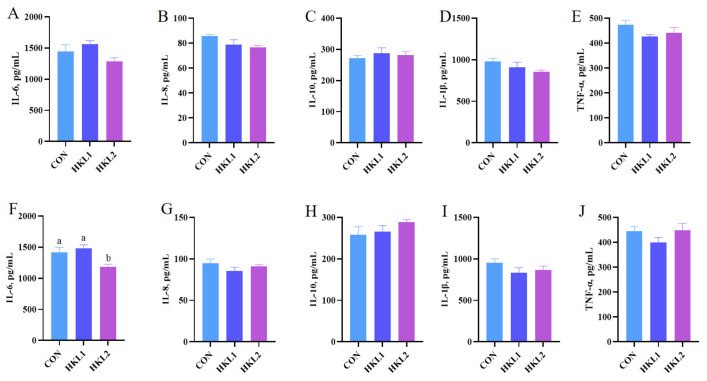
Effects of dietary *K. lactis* hydrolysate (HKL) supplementation on serum inflammatory cytokines in weaned piglets on day 14 (**A**–**E**) and day 28 (**F**–**J**). IL-6, interleukin-6 (**A**,**F**); IL-8, interleukin-8 (**B**,**G**); IL-10, interleukin-10 (**C**,**H**); IL-1β, interleukin-1β (**D**,**I**); TNF-α, tumor necrosis factor-alpha (**E**,**J**). CON = basal diet without additive; HKL1 = CON + HKL (5 g/kg); HKL2 = CON + HKL (10 g/kg). *n* = 6. The result is presented as mean ± standard error. ^a,b^ The value with a different superscript means a significant difference (*p* < 0.05).

As depicted in Figure 5, on day 14, dietary supplementation with HKL had no significant effect on serum inflammatory cytokines in weaned piglets (*p* > 0.05; Figure 5A–E). On day 28, supplementation with 10 g/kg HKL significantly decreased serum IL-6 concentration compared with the CON group (*p* < 0.05; Figure 5F). However, it had no significant effect on the levels of IL-8, IL-10, IL-1β, and TNF-α (*p* > 0.05; Figure 5G–J).

### 3.6. Effects of Dietary HKL on Serum Immunoglobulin Concentrations in Weaned Piglets

**Figure 6:**
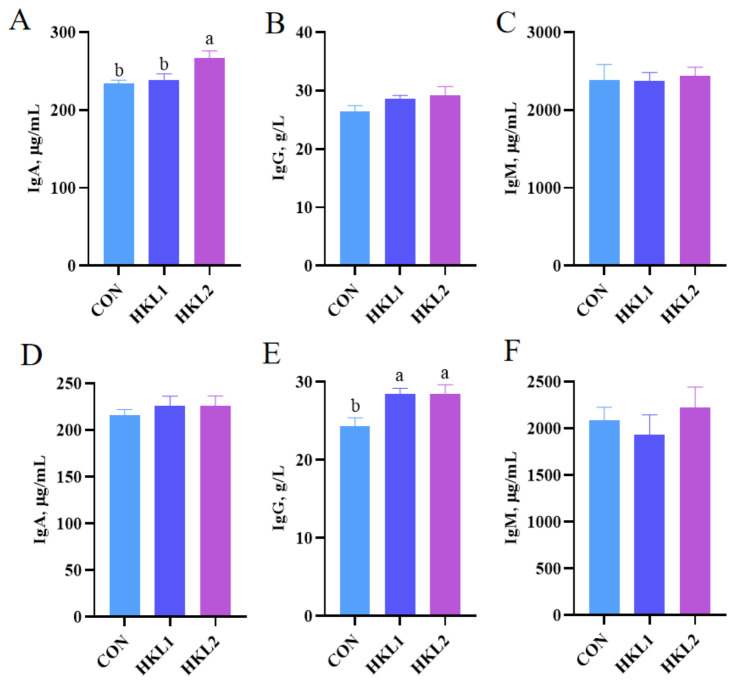
Effects of dietary *K. lactis* hydrolysate (HKL) supplementation on serum immunoglobulin concentrations in weaned piglets on day 14 (**A**–**C**) and day 28 (**D**–**F**). IgA, immunoglobulin A (**A**,**D**); IgG, immunoglobulin G (**B**,**E**); IgM, immunoglobulin M (**C**,**F**). CON = basal diet without additive; HKL1 = CON + HKL (5 g/kg); HKL2 = CON + HKL (10 g/kg). *n* = 6. The result is presented as mean ± standard error. ^a,b^ The value with a different superscript means a significant difference (*p* < 0.05).

As shown in Figure 6, on day 14, supplementation with 10 g/kg HKL significantly increased serum IgA concentration compared with the CON group (*p* < 0.05; Figure 6A). It had no significant effect on IgG and IgM concentrations (*p* > 0.05; Figure 6B,C). On day 28, supplementation with 5 or 10 g/kg HKL significantly increased serum immunoglobulin G (IgG) concentration relative to CON (*p* < 0.05; Figure 6E). However, it had no significant effect on IgA and IgM concentrations (*p* > 0.05; Figure 6D,F).

### 3.7. Effects of Dietary HKL on Fecal Microbiota in Weaned Piglets

**Figure 7:**
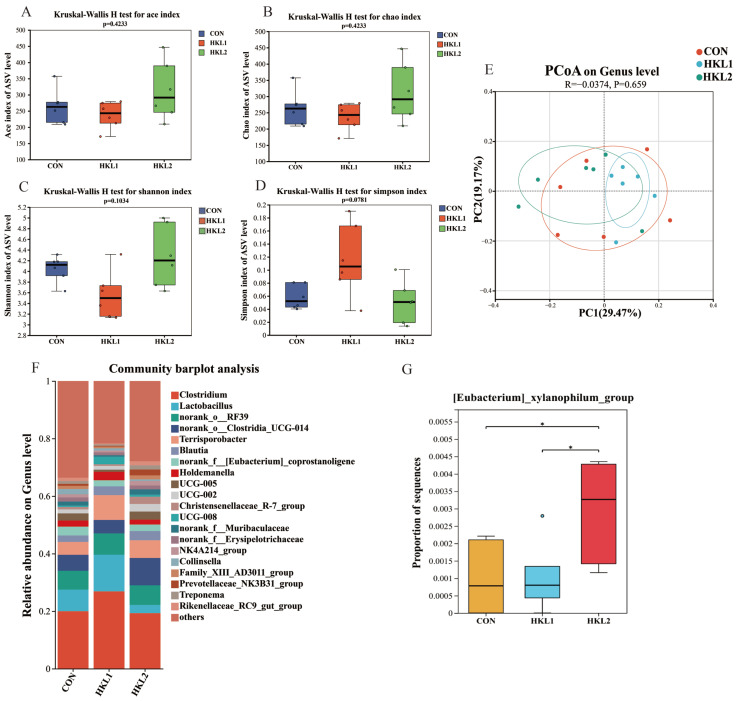
Effects of dietary supplementation with *K*. *lactis* hydrolysate (HKL) on fecal microbial composition of weaned piglets on day 14. α-diversity indices: ACE (**A**), Chao1 (**B**), Shannon (**C**), and Simpson (**D**); Principal coordinate analysis (PCoA) was performed based on Bray–Curtis distance at the genus level (**E**); Genus-level relative abundance profiles (**F**); Differentially abundant genera (**G**). CON = basal diet without additive; HKL1 = CON + HKL (5 g/kg); HKL2 = CON + HKL (10 g/kg). *n* = 6. * indicates a significant adjusted *p*-value.

On day 14, no differences were observed in the α-diversity indices (ACE, Chao, Shannon, and Simpson) among the CON, HKL1 (5 g/kg), and HKL2 (10 g/kg) groups (*p* > 0.05; Figure 7A–D), indicating that HKL supplementation did not markedly affect microbial richness or diversity at this time point. As shown in Figure S1A, principal coordinate analysis (PCoA) at the phylum level further indicated no significant differences in microbial community structure among the different treatment groups (*p* = 0.067, R = –0.1173). Similarly, at the genus level, PCoA showed no significant separation of microbial community structure among treatments (*p* = 0.659, R = –0.0374), and the clustering of samples was generally consistent across groups (Figure 7E).

At the phylum level, the top 6 dominant taxa across all groups were Bacillota, Bacteroidota, Actinomycetota, Pseudomonadota, Spirochaetota, and Chlamydiota (Figure S1C). The relative abundances of Actinomycetota, Pseudomonadota, and Chlamydiota were higher in the CON group than in the HKL1 and HKL2 groups. The relative abundances of Bacillota were higher in the HKL1 group than in the CON and HKL2 groups, and the relative abundances of Bacteroidota and Spirochaetota were higher in the HKL2 group than in the CON and HKL1 groups (Figure S1C). The relative abundance of Bacillota was significantly higher in the HKL1 group compared with the CON group (*p* < 0.05; Figure S1E). At the genus level, the top 10 dominant taxa across groups were *Clostridium*, *Lactobacillus*, norank_o_RF39, norank_o_Clostridia_UCG-014, *Terrisporobacter*, *Blautia*, norank_f_[*Eubacterium*] _*coprostanoligenes*_group, *Holdemanella*, UCG-005, and UCG-002. The relative abundances of *Clostridium*, *Lactobacillus*, norank_o_RF39, *Terrisporobacter*, and *Holdemanella* were higher in HKL1 than in CON and HKL2, whereas norank_o_Clostridia_UCG-014, *Blautia*, UCG-005, and UCG-002 were higher in HKL2 than in CON and HKL1. In contrast, norank_f_[*Eubacterium*]_*coprostanoligenes*_group was significantly more abundant in CON than in HKL1 and HKL2 (Figure 7F). Compared with CON and HKL1, HKL2 significantly increased the relative abundances of [*Eubacterium*]_*xylanophilum*_group (*p* < 0.05; Figure 7G).

**Figure 8:**
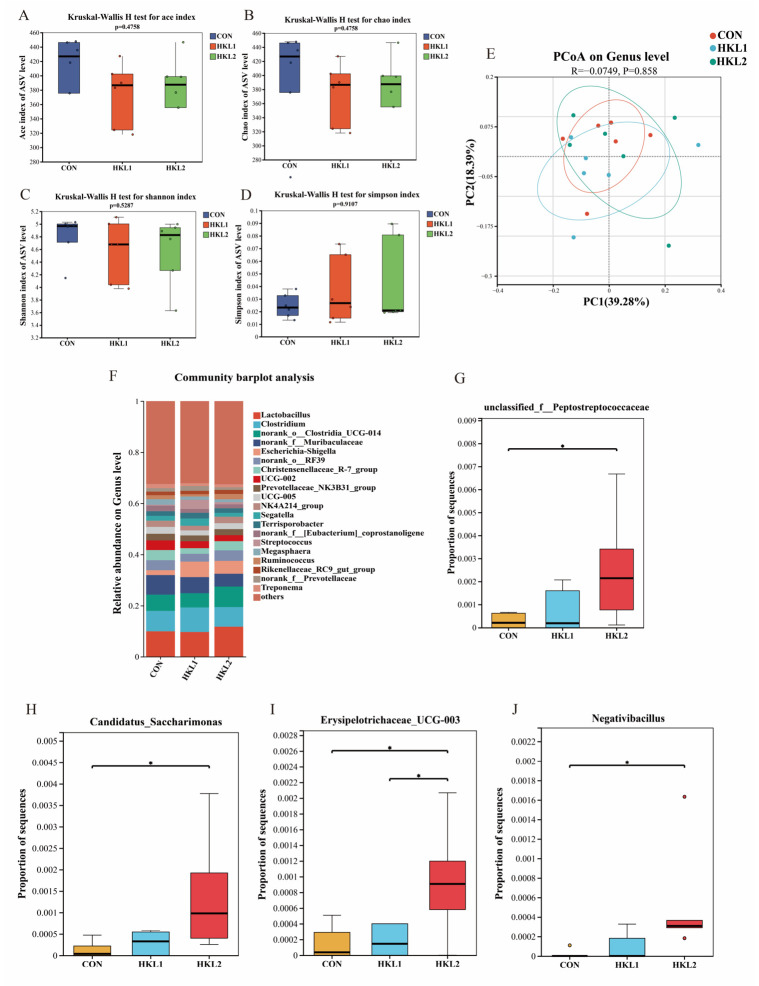
Effects of dietary supplementation with *K*. *lactis* hydrolysate (HKL) on fecal microbial composition of weaned piglets at day 28. α-diversity indices: ACE (**A**), Chao1 (**B**), Shannon (**C**), and Simpson (**D**); Principal coordinate analysis (PCoA) was performed based on Bray–Curtis distance at the genus level (**E**); Genus-level relative abundance profiles (**F**); Differentially abundant genera (**G**–**J**). CON = basal diet without additive; HKL1 = CON + HKL (5 g/kg); HKL2 = CON + HKL (10 g/kg). *n* = 6. * indicates a significant adjusted *p*-value.

On day 28, analysis of microbial diversity revealed that the α-diversity indices (ACE, Chao, Shannon, and Simpson) did not differ between the CON group and the HKL1 or HKL2 groups (*p* > 0.05), indicating that HKL supplementation did not significantly affect fecal microbial richness or diversity throughout the trial (Figure 8A–D). As shown in Figure S1B, PCoA at the phylum level revealed no significant differences in microbial community structure among the different treatment groups (*p* = 0.604, R = –0.028). Genus-level PCoA further revealed no significant differences in community structure among treatments (*p* = 0.858, R = −0.0749), with no clear separation of samples between the HKL-supplemented groups and CON (Figure 8E).

At the phylum level, the top 6 dominant taxa across groups were Bacillota, Bacteroidota, Actinomycetota, Pseudomonadota, Spirochaetota, and Cyanobacteriota (Figure S1D). The relative abundance of Bacteroidota, Spirochaetota, and Actinomycetota was higher in the CON group than in the HKL1 and HKL2 groups. In addition, the relative abundance of Pseudomonadota was higher in the HKL1 group than in the CON and HKL2 groups. In addition (Figure S1D). The relative abundance of Cyanobacteriota was significantly higher in the HKL2 group than in the CON group (Figure S1F). Genus-level composition analysis showed that the top 10 dominant genera across groups were *Lactobacillus*, *Clostridium*, norank_o_Clostridia_UCG-014, norank_f_Muribaculaceae, *Escherichia–Shigella*, norank_o_RF39, Christensenellaceae_R-7_group, UCG-002, Prevotellaceae_NK3B31_group, and UCG-005 (Figure 8F). The relative abundances of *Clostridium* and *Escherichia–Shigella* were higher in HKL1 than in CON and HKL2, whereas *Lactobacillus*, norank_o_Clostridia_UCG-014, and norank_o_RF39 were higher in HKL2 than in CON and HKL1. In contrast, norank_f_Muribaculaceae, Christensenellaceae_R-7_group, UCG-002, Prevotellaceae_NK3B31_group, and UCG-005 were higher in CON than in HKL1 and HKL2 (Figure 8F). Kruskal–Wallis H tests indicated that, compared with CON, HKL2 significantly increased the relative abundances of unclassified_ f_Peptostreptococcaceae, *Candidatus Saccharimonas*, Erysipelotrichaceae_UCG-003, and *Negativibacillus* (*p* < 0.05; Figure 8G–J). In addition, HKL2 significantly increased Erysipelotrichaceae_UCG-003 relative to HKL1 (*p* < 0.05; Figure 8I).

## 4. Discussion

### 4.1. Dietary HKL Improves Growth Performance and Alleviates Diarrhea in Weaned Piglets

In this study, dietary supplementation with HKL significantly increased ADG during days 1–14 and ADFI during days 1–28 in weaned piglets. While concurrently reducing the incidence of diarrhea throughout the 28-day trial. These outcomes corroborate existing literature on the benefits of yeast derivatives. For example, Boontiam et al. [26] documented a linear increase in ADG corresponding with escalating levels of hydrolyzed yeast (0%, 5%, and 10%), which was accompanied by improved CP digestibility and intestinal morphology. Similarly, Shi and Kim reported that a yeast-based composite containing β-glucan, mannans, nucleotides, and exogenous enzymes boosted both ADG and ADFI [27]. The growth promotion observed in the current investigation is likely attributable to the highly digestible proteins, small peptides, and nucleotides present in HKL, which may enhance nutrient utilization and support intestinal function in weaned piglets [26]. Specifically, our data revealed that HKL significantly increased the apparent digestibility of DM, ash, calcium, and phosphorus, thereby providing direct evidence of improved nutrient utilization during the critical post-weaning phase. Furthermore, given that diarrhea is a primary constraint on growth post-weaning, the reduction in diarrhea incidence—paralleling findings for brewer’s yeast by Trckova et al. [28]—suggests that HKL effectively mitigates weaning stress by supporting intestinal function and resilience.

### 4.2. Dietary HKL Specifically Promotes the ATTD of Minerals

Results from this study demonstrated that HKL supplementation significantly improved the ATTD of DM, ash, calcium, and phosphorus, whereas no significant effects were observed for CP, EE, or GE. This distinct profile implies that the beneficial impacts of HKL are likely mediated through mechanisms targeting intestinal mucosal integrity and mineral bioavailability, rather than through the provision of broad-spectrum digestive enzymes. The elevation in DM digestibility indicates a general enhancement of the piglets’ digestive and absorptive capacity, potentially driven by bioactive components in HKL such as nucleotides and mannan-oligosaccharides (MOS), which are known to preserve intestinal morphology [6]. Bioactive components in HKL, such as nucleotides and MOS, have been reported to support intestinal development and nutrient utilization in weaned piglets [29]. However, intestinal morphology was not evaluated in the present study, and the mechanisms underlying the improved digestibility require further investigation. Nevertheless, the moderate improvement in DM digestibility suggests that the absorption of specific nutrients—particularly CP and EE—remains constrained by factors beyond surface area.

It is worth noting that the concurrent increase in ash, calcium, and phosphorus digestibility, absent a corresponding rise in CP digestibility, presents a compelling finding. We hypothesize that this phenomenon is linked to the degradation of phytate-mineral complexes. Phytic acid in plant-based feedstuffs chelates minerals, obstructing their absorption [30]. One possible explanation is that bioactive components released during yeast hydrolysis may have improved the intestinal environment and nutrient absorption capacity, thereby facilitating mineral utilization [31,32,33]. This mineral liberation accounts for the observed rise in ash digestibility. Supporting this, previous studies have reported that yeast proteins improve calcium absorption in rats [34], while Matsui et al. [35] demonstrated the efficacy of yeast-derived phytase in improving phosphorus bioavailability in swine diets.

Conversely, the stagnation in CP and EE digestibility suggests that HKL does not supply sufficient exogenous protease or lipase activity to compensate for the endogenous enzyme deficiencies typical in weaned piglets. Although small peptides and amino acids in HKL may support gut health, they appear insufficient to assist in the hydrolysis of complex dietary proteins. Furthermore, fat digestion in piglets is primarily limited by inadequate bile acid secretion rather than a lack of luminal lipase [36]. Since HKL lacks bile salts, it cannot address this specific physiological limitation. Consequently, with the digestibility of CP and EE—primary determinants of dietary energy density—remaining unchanged, the ATTD of GE was unaffected. In summary, while HKL improves DM and mineral digestibility likely via morphological modulation and mineral chelation, its inability to enhance CP and EE digestibility distinguishes its mode of action from that of exogenous enzyme additives. Future studies exploring co-supplementation (e.g., with proteases or emulsifiers) may be necessary to fully harness HKL’s potential for energy and protein utilization.

### 4.3. Dynamic Changes in Blood Biochemical Indices

Blood biochemical indices serve as indicators of the animal’s metabolic status in response to nutrient intake. On day 14, serum levels of TP and GLU were significantly elevated in the HKL group. Higher GLU levels typically reflect an improved energy status [37], while elevated TP suggests enhanced protein nutrition and utilization [38], aligning with the observed growth performance. While some studies have reported negligible effects of yeast products on lipid or protein indices [39], such variability likely reflects strain-specific compositional differences. Notably, by day 28, these inter-group differences had dissipated. This temporal pattern suggests that the regulatory effects of HKL are most critical during the early post-weaning stage (day 14) when stress is most acute. As the piglets mature and their digestive and immune systems develop, the metabolic status of the control group naturally recovers, thereby diminishing the differential effects of the treatment.

### 4.4. Enhancement of Antioxidant Capacity to Mitigate Oxidative Stress

Weaning stress frequently exacerbates oxidative damage in piglets [40]. In the present study, HKL supplementation significantly increased the activities of SOD and GSH-Px while reducing MDA concentrations. Interestingly, the upregulation of SOD and GSH-Px was more pronounced during the later stage (day 28). These findings indicate that HKL plays a vital role in fortifying the antioxidant defense system and curbing lipid peroxidation, consistent with previous reports on brewer’s yeast [41] and yeast polysaccharides [42]. Although some studies have noted MDA reduction without concurrent changes in antioxidant enzymes [26,42], such discrepancies may be attributed to variations in cell wall composition, metabolites, or microbiota modulation among yeast strains, which can influence signaling pathways such as nuclear factor erythroid 2-related factor 2/antioxidant response element (Nrf2/ARE) [43]. Overall, HKL effectively alleviated weaning-induced oxidative damage by boosting antioxidant enzyme activities, thereby supporting a healthy growth trajectory.

### 4.5. Modulation of Systemic Inflammation and Enhancement of Humoral Immunity

Weaned piglets are highly vulnerable to stress-induced compromise of the intestinal barrier, immune dysfunction, and inflammation, all of which can hinder growth [44]. Here, HKL supplementation significantly reduced plasma concentrations of the pro-inflammatory cytokine IL-6 on day 28, indicating an alleviation of systemic inflammation. This aligns with reports that selenium-enriched yeast enhances feed efficiency and lowers IL-6 in swine [45]. A plausible mechanism is that HKL improves systemic redox balance; enhanced antioxidant defenses are frequently associated with diminished production of pro-inflammatory cytokines [46]. Furthermore, HKL modulated immunoglobulin levels, increasing IgA on day 14 and IgG on day 28. As critical components of disease resistance, elevated IgA and IgG reflect an enhanced humoral immune status. These results corroborate previous findings that yeast postbiotics [47] and live yeast [48] elevate serum immunoglobulins. While certain studies have reported no changes in IgG or immune indices following yeast supplementation [49,50], these inconsistencies likely stem from differences in cell wall structure and bioavailability. Collectively, HKL improved the health status of weaned piglets by attenuating systemic inflammation and bolstering humoral immunity.

### 4.6. Regulation of Gut Microbiota Structure to Promote Intestinal Health

The gut microbiota is instrumental in maintaining animal health [51]. The porcine gut harbors a dense microbial community, with 10^10^ to 10^11^ microorganisms per gram of content [52], which governs digestion, disease resistance, and metabolite synthesis [53,54]. Although HKL did not significantly alter the alpha diversity or overall community structure (PCoA) of fecal microbiota in this study, it induced significant shifts in specific taxa, which may underpin its probiotic mechanisms. On day 14, HKL significantly increased the relative abundance of the [*Eubacterium*]_*xylanophilum*_group. This group is known to enhance the anti-inflammatory cytokine IL-10 and correlates positively with serum antioxidant enzymes (GSH-Px, CAT) and negatively with MDA [55], mirroring the antioxidant improvements observed herein. Additionally, HKL enriched the genus Saccharimonas, which has been shown to inhibit inflammatory responses by binding to immune cell co-receptors [56] and to alleviate colitis by increasing organic acid concentrations [57].

Moreover, HKL increased the abundance of unclassified_f_Peptostreptococcaceae and *Negativibacillus*, genera known for their capacity to produce short-chain fatty acids (SCFAs). Accumulating evidence indicates that SCFAs can help maintain the intestinal barrier and modulate redox balance [58,59]. In summary, dietary HKL modulated the intestinal microecology of weaned piglets by selectively enriching beneficial genera (e.g., acid-producing and anti-inflammatory bacteria), which may contribute to improved barrier function and anti-inflammatory effects. Further studies are needed to confirm whether SCFA or other organic acid production was affected.

## 5. Conclusions

Dietary supplementation with HKL improved feed intake and early growth performance and showed a tendency to increase overall ADG in weaned piglets. Furthermore, HKL enhanced the digestibility of calcium and phosphorus while fostering a more favorable immune-antioxidant status and reducing systemic inflammation. Overall, HKL emerges as a promising functional feed additive for weaned piglet diets, with a dosage of 10 g/kg demonstrating superior efficacy.

## Figures and Tables

**Table 1:** The composition of the diet and nutrient levels (as-fed basis).

Items	Prestarter Days 1~14			Starter Days 15~28		
	CON	HKL1	HKL2	CON	HKL1	HKL2
Ingredients, %						
Corn	35.81	35.51	35.31	50.86	50.61	50.36
Expanded corn	15.00	15.00	15.00	10.00	10.00	10.00
Soybean meal (46% crude protein)	16.80	16.60	16.30	19.00	18.75	18.50
Expanded soybean	13.50	13.50	13.50	8.50	8.50	8.50
Fish meal	3.00	3.00	3.00	1.00	1.00	1.00
Whey powder	10.00	10.00	10.00	3.00	3.00	3.00
Bran	0.00	0.00	0.00	1.50	1.50	1.50
Soybean oil	1.00	1.00	1.00	1.40	1.40	1.40
Calcium phosphate	0.70	0.70	0.70	0.88	0.88	0.88
Limestone (CaCO_3_)	1.00	1.00	1.00	1.06	1.06	1.06
NaCl	0.40	0.40	0.40	0.40	0.40	0.40
Choline chloride (60%)	0.05	0.05	0.05	0.05	0.05	0.05
L-Lysine HCL (78.8%)	1.15	1.15	1.15	1.02	1.02	1.02
DL-Methionine (99%)	0.09	0.09	0.09	0.08	0.08	0.08
L-Threonine (98.5%)	0.26	0.26	0.26	0.23	0.23	0.23
L-Tryptophan (98%)	0.02	0.02	0.02	0.00	0.00	0.00
Phytase ^1^	0.02	0.02	0.02	0.02	0.02	0.02
Zinc oxide	0.20	0.20	0.20	0.00	0.00	0.00
*K. lactis* hydrolysate	0.00	0.50	1.00	0.00	0.50	1.00
Premix ^2^	1.00	1.00	1.00	1.00	1.00	1.00
Total	100.00	100.00	100.00	100.00	100.00	100.00
Analyzed nutrient content, %						
Crude protein	19.59	19.24	19.24	18.46	18.16	18.42
Calcium	1.10	1.12	1.17	1.08	1.07	1.10
Phosphorus	0.67	0.58	0.56	0.56	0.50	0.47
Ether extract	5.48	5.41	5.97	5.73	5.24	5.64
Crude ash	6.21	6.19	6.54	6.22	6.02	6.29
Gross energy, kcal/kg	3985.96	3990.02	4035.35	3955.93	3976.52	4012.40
Calculated nutrient content ^3^, %						
Metabolizable energy, kcal/kg	3399.12	3400.85	3402.68	3354.24	3356.02	3357.80
SID Lysine	1.30	1.30	1.30	1.15	1.15	1.15
SID Methionine	0.38	0.38	0.38	0.34	0.34	0.34
SID Threonine	0.76	0.76	0.76	0.68	0.68	0.68
SID Tryptophan	0.22	0.22	0.22	0.19	0.19	0.19

SID = Standardized ileal digestibility. ^1^ 1 × 10^4^ phytase unit per kg of products (Inner Mongolia Yiduoli Biotechnology Co., Ltd., Hohhot, China). ^2^ Premix supplied per kg of diet: vitamin A, 35.2 mg; vitamin D_3_, 7.68 mg; DL-α-tocopherol, 128 mg; menadione sodium bisulfite, 8.16 mg; thiamine mononitrate, 4 mg; riboflavin, 12 mg; pyridoxine hydrochloride, 8.32 mg; cyanocobalamin, 4.8 mg; niacin, 38.4 mg; calcium pantothenate, 25 mg; folic acid, 1.68 mg; biotin, 0.16 mg; iron (FeSO_4_·H_2_O), 171 mg; manganese (MnSO_4_·H_2_O), 42.31 mg; copper (CuSO_4_·5H_2_O), 125 mg; selenium (Na_2_SeO_3_), 0.19 mg; cobalt (CoCl_2_), 0.19 mg; iodine (Ca(IO_3_)_2_), 0.54 mg. ^3^ The calculated nutrient contents were derived from the composition values of feed ingredients, according to the National Research Council (NRC, 2012) [24].

**Table 2:** Comparison of free amino acid levels before and after hydrolysis of *K*. *lactis* (mg/L).

Items	Before Hydrolysis	After Hydrolysis	SEM	*p*-Value
Aspartic acid (Asp)	226.59	1497.56	65.708	0.010
Glutamic acid (Glu)	1153.43	6689.68	362.969	0.016
Serine (Ser)	116.91	1961.88	111.432	0.014
Histidine (His)	338.53	829.38	29.467	0.013
Glycine (Gly)	170.00	691.72	40.519	0.018
Threonine (Thr)	210.53	1014.76	15.984	0.001
Arginine (Arg)	1827.52	3482.70	236.811	0.066
Alanine (Ala)	798.89	5595.58	329.313	0.017
Tyrosine (Tyr)	432.92	2153.41	134.299	0.020
Valine (Val)	799.94	2795.83	171.243	0.027
Methionine (Met)	150.11	924.16	54.502	0.015
Norvaline (Nva)	10.00	10.00	0.000	1.000
Isoleucine (Ile)	337.62	1807.16	102.743	0.018
Phenylalanine (Phe)	131.94	1840.07	108.926	0.016
Lysine (Lys)	1446.58	2893.99	192.818	0.061
Leucine (Leu)	208.44	3213.40	194.265	0.016
Total free amino acids	8359.98	37,401.28	65.708	0.020

**Table 3:** Nutritional components of HKL (air dry basis, %).

Items	Composition
Dry matter	93.97
Ether extract	1.70
Crude protein	33.05
Trichloroacetic acid-soluble protein	9.19
Mannan	7.18
β-glucan	10.90

**Table 4:** Effect of dietary *K. lactis* hydrolysate (HKL) supplementation on growth performance of weaned piglets ^1^.

Items	Experimental Groups			SEM	*p*-Value
	CON	HKL1	HKL2		
BW, kg					
Day 0	6.07	6.07	6.04	0.154	0.964
Day 14	8.73 ^y^	8.86 ^y^	9.53 ^x^	0.259	0.089
Day 28	13.44	14.08	13.86	0.751	0.745
ADG, g					
Day 1–14	180.20 ^b^	198.85 ^b^	248.76 ^a^	14.546	0.015
Day 15–28	298.11	373.89	309.68	36.514	0.105
Day 1–28	228.14 ^y^	286.37 ^x^	279.23 ^x^	23.397	0.059
ADFI, g					
Day 1–14	286.47 ^b^	323.33 ^ab^	338.77 ^a^	17.386	0.120
Day 15–28	553.02	637.58	595.48	44.335	0.183
Day 1–28	389.50 ^b^	480.46 ^a^	467.12 ^a^	31.877	0.043
F: G					
Day 1–14	1.62 ^a^	1.63 ^a^	1.37 ^b^	0.061	0.031
Day 15–28	1.99	1.72	1.98	0.129	0.305
Day 1–28	1.76	1.68	1.70	0.086	0.859

BW = body weight; ADG = average daily gain; ADFI = average daily feed intake; F: G = feed-to-gain ratio. ^1^ CON = basal diet without additive; HKL1 = CON + HKL (5 g/kg); HKL2 = CON + HKL (10 g/kg). *n* = 6. ^a,b^ The value with a different superscript means a significant difference (*p* < 0.05). ^x,y^ The value with a different superscript means a tendency to be different (0.05 ≤ *p* < 0.10).

**Table 5:** Effect of dietary *K*. *lactis* hydrolysate (HKL) supplementation on diarrhea incidence of weaned piglets ^1^.

Items	Experimental Groups			*p*-Value
	CON	HKL1	HKL2	
Day 1–14	41.60 ^a^	25.00 ^b^	26.20 ^b^	<0.001
Day 15–28	17.00 ^a^	7.90 ^b^	9.90 ^b^	<0.001

^1^ CON = basal diet without additive; HKL1 = CON + HKL (5 g/kg); HKL2 = CON + HKL (10 g/kg). *n* = 6. ^a,b^ The value with different superscripts means a significant difference (*p* < 0.05).

## Data Availability

All data used for this study appear in the illustrated figures, and the raw data will promptly be made available upon request.

## Supplementary Materials

The following supporting information can be downloaded at: https://www.mdpi.com/article/10.3390/microorganisms14071440/s1, Figure S1: Effects of dietary supplementation with *K. lactis* hydrolysate (HKL) on fecal microbial composition at the phylum level of weaned piglets.

## Abbreviations

The following abbreviations are used in this manuscript:

ADF	Acid detergent fiber
ALT	Alanine aminotransferase
ALB	Albumin
AST	Aspartate aminotransferase
ADFI	Average daily feed intake
ADG	Average daily gain
AIA	Acid-insoluble ash
AOAC	Association of Official Analytical Chemists
ASV	Amplicon sequence variants
ATTD	Apparent total tract digestibility
CAT	Catalase
CF	Crude fiber
CP	Crude protein
DM	Dry matter
F: G	Feed-to-gain ratio
GE	Gross energy
GLU	Glucose
GSH-Px	Glutathione peroxidase
HDL-C	High-density lipoprotein cholesterol
HKL	Kluyveromyces lactis hydrolysate
IgA	Immunoglobulin A
IgG	Immunoglobulin G
IgM	Immunoglobulin M
IL-6	Interleukin-6
LDL-C	Low-density lipoprotein cholesterol
MDA	Malondialdehyde
PCoA	Principal coordinate analysis
SOD	Superoxide dismutase
T-AOC	Total antioxidant capacity
TC	Total cholesterol
TG	Triglyceride
TP	Total protein
V/C	Villus height-to-crypt depth ratio
