# Supplementary material for: Kluyveromyces lactis Hydrolysate Enhances Growth Performance and Reduces Diarrhea Rate by Modulating Immune Function and Regulating Gut Microbiota in Weaned Piglets
Source: Microorganisms. 2026 Jun 30;14(7):1440. doi: 10.3390/microorganisms14071440 (PMC13413709; doi:10.3390/microorganisms14071440)
Supplement: Supplementary file 1 [file microorganisms-14-01440-s001.zip › microorganisms-4356516-supplementary.pdf]

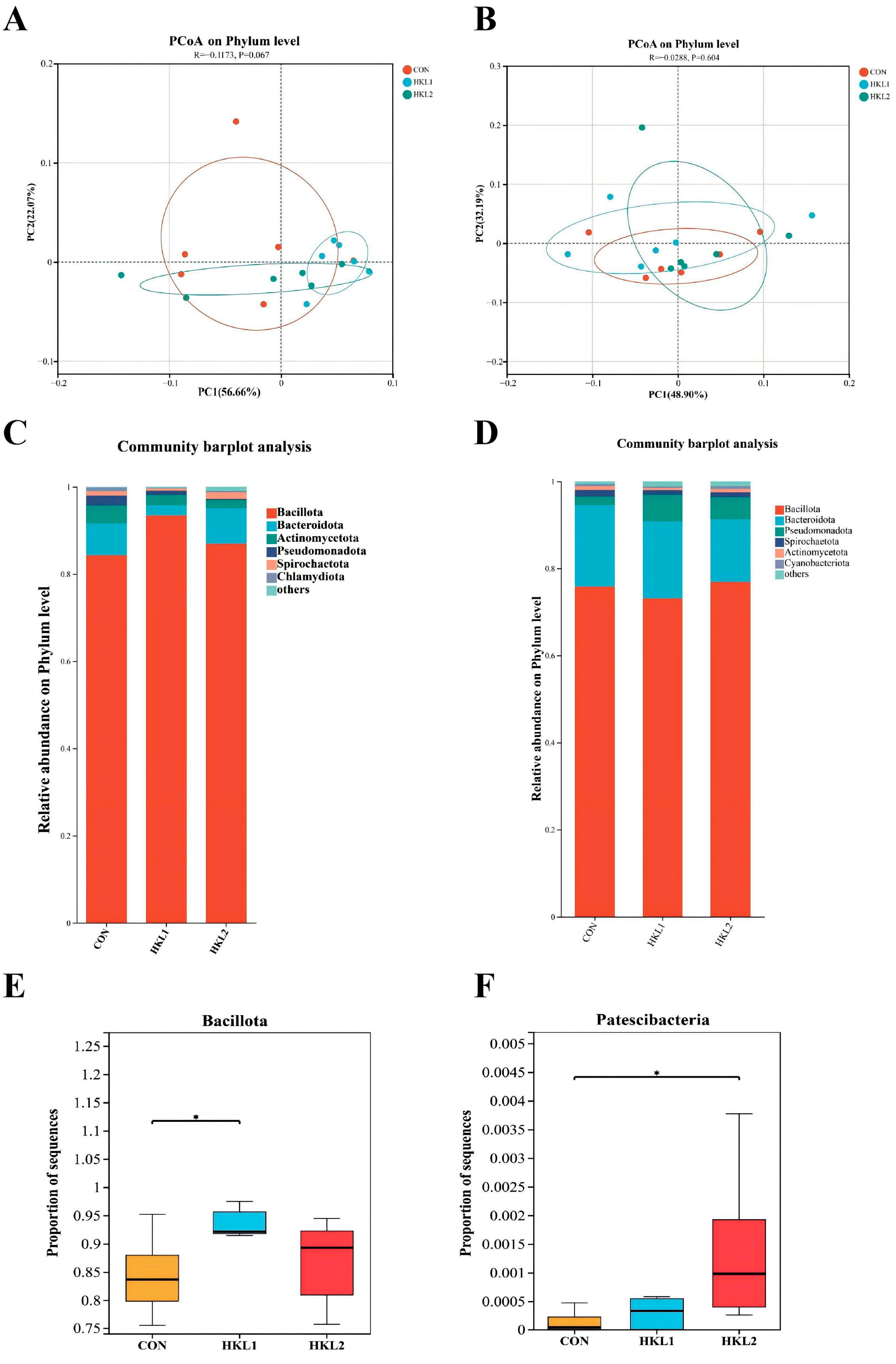

Figure S1. Effects of dietary supplementation with *K. lactis* hydrolysate (HKL) on fecal microbial composition at the phylum level of weaned piglets. Principal coordinate analysis (PCoA) was performed based on Bray-curtis

distance at the phylum level on day 14 (**A**) and day 28 (**B**); Phylum-level relative abundance profiles on day 14 (**C**) and day 28 (**D**); Differentially abundant genera on day 14 (**E**) and day 28 (**F**). CON = basal diet without additive; HKL1 = CON + HKL (5 g/kg); HKL2 = CON + HKL (10 g/kg). *n* = 6. * indicates a significant adjusted p-value.
